# High correlation between CT and clinical data in COVID‐19 progression

**DOI:** 10.1002/jcu.23163

**Published:** 2022-03-12

**Authors:** Tommaso D'Angelo, Christian Booz

**Affiliations:** ^1^ Department of Biomedical Sciences and Morphological and Functional Imaging University Hospital Messina Messina Italy; ^2^ Department of Radiology and Nuclear Medicine Erasmus MC Rotterdam The Netherlands; ^3^ Division of Experimental Imaging, Department of Diagnostic and Interventional Radiology University Hospital Frankfurt Frankfurt am Main Germany

In this interesting retrospective study, the authors correlate severity scores in computed tomography lung scans with the nasopharyngeal viral load as well as further laboratory data in 37 patients suffering from COVID‐19. In their study, the authors divided patients into non‐severely ill and severely ill patients. The authors classified the severity of the participants' disease as follows: (1) a mildly ill group, in which patients exhibited mild clinical symptoms and no imaging manifestations of pneumonia; (2) a moderately ill group, in which patients had a fever and respiratory symptoms, and signs of pneumonia were observed in imaging; (3) a severely ill group, in patients exhibited dyspnea, a respiratory rate ≥ 30 breaths/min, oxygen saturation (SpO2) ≤93% at a resting state, or arterial partial pressure of oxygen/oxygen concentration (FiO2) ≤300 mm Hg; pulmonary imaging showed marked lesion progression >50% within 24–48 h; (4) a critically ill group, in which patients who had any of the following symptoms had to be admitted to the intensive care unit: respiratory failure and requiring mechanical ventilation, or shock combined with the failure of other organs. Patients who were severely and critically ill were labeled “severely ill patients,” while mildly/moderately ill patients were defined as “not severely ill patients.” Classification of chest computed tomography (CT) severity was primarily based on the invasion range and morphological characteristics of lesions as follows: CT‐1, ground glass opacities (GGO) in ≤25% of the lung parenchyma; CT‐2, GGO in 26%–50% of the lung parenchyma; CT‐3, GGO and consolidation in 51%–75% of the lung parenchyma; CT‐4, GGO and consolidation in ≥76% of the lung parenchyma. The extent of lesions in each lung lobe was evaluated applying a score ranging from 0 to 4.^1^, ^2^ A summation of the scores from all five lobes provided the total CT severity score ranging from 0 to 20. In this context, it should be noted that it remains unclear if dedicated reconstructions such as minimum intensity projection series were applied, since these reconstructions can significantly improve the visualization and detection of GGO compared to standard CT reconstructions, particularly in the initial period of disease.^3^

The idea to correlate CT severity scores with clinical and laboratory data is not new, however, there are only few studies evaluating correlation in the context of dynamic disease progression.^4^, ^5^ The results show that in the case of COVID‐19 progression nasopharyngeal viral load and lung CT severity were closely related, and lung CT severity negatively correlated with lymphocyte and mononuclear cell counts, as well as cycle threshold (Ct) values of largely coronavirus‐expressed genes. Among severely ill COVID‐19 patients, the Ct value was negatively related to CRP and CT severity but did not correlate with lymphocyte, mononuclear cell, neutrophil counts, procalcitonin, and length of hospital stay. The authors concluded that an increased viral load and CRP were associated with increased lung inflammation, which has been demonstrated by studies in the literature.^6^ When the severity of the disease progressed, lymphocyte counts decreased but no statistical correlation was observed between lymphocyte counts and Ct values. The results also showed that CT severity negatively correlated with lymphocyte and mononuclear cell counts. At the critical stage, both viral infection and inflammatory factors are involved in lung inflammation, and corresponding treatment strategies should be adopted to avoid the occurrence of cytokine storm and the development of ARDS in patients.^7^

The authors concluded that by closely monitoring CT, clinical, and laboratory indicators, early intervention measures should be taken to reduce the occurrence of mortality. We fully agree with this hypothesis, however, some questions remain regarding the value of CT imaging COVID‐19. What is the clinical benefit of CT scanning of the lungs in patients compared to patients only having clinical and laboratory data? Obviously, CT angiography should be performed to exclude complications such as pulmonary embolism or bleeding. However, which is the indication for unenhanced CT imaging of the lungs in COVID‐19? When should an unenhanced CT of the lungs be performed and when clinical and laboratory data are enough? Based on the knowledge of high correlations between CT severity and clinical and laboratory data in case of progression, these questions might be answered in future studies.

In summary, we agree with the authors' hypothesis that CT severity scoring in combination with clinical and laboratory data should be applied for disease and therapy monitoring in COVID‐19, particularly in the case of progression. However, the isolated value of CT scanning of the lungs remains unclear and should be furtherly investigated.

## Data Availability

Data sharing is not applicable to this article as no new data were created or analyzed in this study.

## References

[1] Zhang R , Ouyang H , Fu L , et al. CT features of SARS‐CoV‐2 pneumonia according to clinical presentation: a retrospective analysis of 120 consecutive patients form Wuhan city. Eur Radiol. 2020;30:4417‐4426. 32279115 10.1007/s00330-020-06854-1 PMC7150608

[2] Li K , Fang Y , Li W , et al. CT image visual quantitative evaluation and clinical classification of coronavirus disease (COVID‐19). Eur Radiol. 2020;30:4407‐4416. 32215691 10.1007/s00330-020-06817-6 PMC7095246

[3] Booz C , Vogl TJ , Schoepf UJ , et al. Value of minimum intensity projections for chest CT in COVID‐19 patients. Eur J Radiol. 2021;135:109478. 33360269 10.1016/j.ejrad.2020.109478 PMC7831963

[4] Mader C , Bernatz S , Michalik S , et al. Quantification of COVID‐19 opacities on chest CT–evaluation of a fully automatic AI‐approach to noninvasively differentiate critical versus noncritical patients. Acad Radiol. 2021;28(8):1048‐1057. 33741210 10.1016/j.acra.2021.03.001 PMC7936551

[5] Chun‐Shuang G , Zhi‐Bin L , Jing‐Jing L , et al. CT appearances, patterns of progression, and follow‐up of COVID‐19: evaluation on thin‐section CT. Insights Imaging. 2021;12:73. 34110540 10.1186/s13244-021-01019-0 PMC8190726

[6] Fajnzylber J , Regan J , Coxen K , et al. SARS‐CoV‐2 viral load is associated with increased disease severity and mortality. Nat Commun. 2020;11:5493. 33127906 10.1038/s41467-020-19057-5 PMC7603483

[7] Zheng L , Fang SH , Jun‐Xia L , et al. Clinical efficacy of corticosteroids in the early stages of deterioration in COVID‐19 pneumonia. Infect Drug Resist. 2021;14:2667‐2674. 34285519 10.2147/IDR.S314938 PMC8285565

